# Predictive Genes in Adjacent Normal Tissue Are Preferentially Altered by sCNV during Tumorigenesis in Liver Cancer and May Rate Limiting

**DOI:** 10.1371/journal.pone.0020090

**Published:** 2011-07-05

**Authors:** John R. Lamb, Chunsheng Zhang, Tao Xie, Kai Wang, Bin Zhang, Ke Hao, Eugene Chudin, Hunter B. Fraser, Joshua Millstein, Mark Ferguson, Christine Suver, Irena Ivanovska, Martin Scott, Ulrike Philippar, Dimple Bansal, Zhan Zhang, Julja Burchard, Ryan Smith, Danielle Greenawalt, Michele Cleary, Jonathan Derry, Andrey Loboda, James Watters, Ronnie T. P. Poon, Sheung T. Fan, Chun Yeung, Nikki P. Y. Lee, Justin Guinney, Cliona Molony, Valur Emilsson, Carolyn Buser-Doepner, Jun Zhu, Stephen Friend, Mao Mao, Peter M. Shaw, Hongyue Dai, John M. Luk, Eric E. Schadt

**Affiliations:** 1 Rosetta Inpharmatics, LLC, a wholly owned subsidiary of Merck & Co., Inc., Seattle, Washington, United States of America; 2 Merck & Co., Upper Gwynedd, Pennsylvania, United States of America; 3 Merck & Co., Boston, Massachusetts, United States of America; 4 Department of Surgery, The University of Hong Kong, Queen Mary Hospital, Hong Kong, China; 5 Sage Bionetworks, Fred Hutchinson Cancer Research Center, Seattle, Washington, United States of America; National Cancer Institute, United States of America

## Abstract

**Background:**

In hepatocellular carcinoma (HCC) genes predictive of survival have been found in both adjacent normal (AN) and tumor (TU) tissues. The relationships between these two sets of predictive genes and the general process of tumorigenesis and disease progression remains unclear.

**Methodology/Principal Findings:**

Here we have investigated HCC tumorigenesis by comparing gene expression, DNA copy number variation and survival using ∼250 AN and TU samples representing, respectively, the pre-cancer state, and the result of tumorigenesis. Genes that participate in tumorigenesis were defined using a gene-gene correlation meta-analysis procedure that compared AN versus TU tissues. Genes predictive of survival in AN (AN-survival genes) were found to be enriched in the differential gene-gene correlation gene set indicating that they directly participate in the process of tumorigenesis. Additionally the AN-survival genes were mostly not predictive after tumorigenesis in TU tissue and this transition was associated with and could largely be explained by the effect of somatic DNA copy number variation (sCNV) in cis and in trans. The data was consistent with the variance of AN-survival genes being rate-limiting steps in tumorigenesis and this was confirmed using a treatment that promotes HCC tumorigenesis that selectively altered AN-survival genes and genes differentially correlated between AN and TU.

**Conclusions/Significance:**

This suggests that the process of tumor evolution involves rate-limiting steps related to the background from which the tumor evolved where these were frequently predictive of clinical outcome. Additionally treatments that alter the likelihood of tumorigenesis occurring may act by altering AN-survival genes, suggesting that the process can be manipulated. Further sCNV explains a substantial fraction of tumor specific expression and may therefore be a causal driver of tumor evolution in HCC and perhaps many solid tumor types.

## Introduction

A universal feature of cancer cells is genomic instability [1], [2], [3], [4], [5], which is thought to be required to generate sufficient variability from which advantageous changes for tumor growth and survival are selected [6]. Following this paradigm, it is now understood that genomic instability can arise from defects in DNA synthesis and repair, chromosome segregation, checkpoints, telomere loss and other biological processes that result in point mutations, copy number variation and gain/loss of biological functions [2], [3], [4], [7], [8], [9], [10]. Hepatocellular carcinoma (HCC) is the second most prevalent cancer of Asian populations and the third leading cause of cancer death in the world. Currently the only effective treatment option is surgery [11]. HCC commonly arises in patients with viral hepatitis and/or cirrhosis where extensive inflammation exposes hepatocytes to mitogenic stimuli [11]. The pre-neoplastic phase is characterized by a number of changes, including the emergence of telomere shortening and the appearance of genomic alterations [12]. Structural changes in the genome progressively accumulate during the transition to neoplasia and from early to late stage HCC [11]. Genomic alterations in HCC are heterogeneous in that many loci have been reported to be altered but generally at a low prevalence [12]. This leads to the hypothesis that there are alternate perturbations that promote tumorigenesis in HCC [11], [12].

Integrative genomics analysis has been successfully applied to many non-cancer diseases [13], [14] and has described networks of gene variation by testing all possible associations across diverse populations segregating the disease of interest. This work has established that genes are generally part of coherent networks, and that the most significant associations of genes to disease often occur in the context of network sub-regions where many or all members of these sub-networks are associated with each other and with disease traits [13], [15], [16]. Such sub-networks have further been associated with DNA variation and validated as causally driving disease outcome [13].

Here we have examined gene network structure using a collection of ∼250 matched tumor (TU) and adjacent normal (AN) samples removed from HCC patients during surgical resection and have assessed whether these networks are associated with DNA and disease variation in the HCC cohort. The approach was in essence to uncover interactions within and between the data types measured in this population (DNA, expression, survival) in AN and TU tissues in an open ended, comprehensive and completely data driven manner. The interactions characteristic of tumors (TU) were compared to normal (AN) tissue to reveal tumor specific changes. Here we present the results of that comprehensive analysis and show that sCNV robustly alters the expression of a large number of genes and also the relationship of those genes to survival in either AN or TU tissue, and that tumorigenesis largely involves disruption of normal functions and the activation of a smaller set of functions that may be critical to disease progression. The data suggested that genes predictive of survival in AN tissue may be rate limiting steps for tumorigenesis. Consistent with this hypothesis a treatment that induces HCC tumorigenesis in mice, MET oncogene overexpression, was found to selectively alter the expression of genes predictive of survival in AN tissue of humans.

## Results

To characterize gene networks in human liver tumor and adjacent normal tissues we compiled a tissue specific cohort comprised of liver tumor (TU) and adjacent normal (AN) tissues from 272 Asian subjects (including 151 paired TU and AN samples, Supplementary Table S1 [17]) undergoing surgical resection for treatment of HCC. RNA was isolated from all TU and AN samples and profiled on a custom Affymetrix microarray comprised of oligonucleotide probes targeting transcripts representing 37,585 known and predicted genes, including high-confidence non-coding RNA sequences. DNA was isolated from all AN and TU tissues and genotyped on the Illumina 650Y SNP genotyping array. Copy number aberration markers (sCNV markers) were then imputed for 32,711 locations in the genome from this high-density SNP panel (Methods).

### Gene networks in liver tumor and adjacent normal samples

Given the large scale genomic changes generally known to occur in tumor samples, our observation of large-scale expression differences between the AN and TU samples in the HCC cohort was not surprising, with 28,233 (>75%) of the 37,585 genes represented on the microarray used in this study detected as differentially expressed (p<0.05, FDR 0.07, see Table S2), consistent with previous reports [18]. The main problem then in interpreting molecular state changes between AN and TU is distinguishing between those changes that are relevant to the progression of the tumor from those changes that are simply tracking with the genomic changes but not relevant to the tumor biology. Therefore, we sought to characterize the impact these large-scale changes had on the connectivity structure of the tissue-specific gene networks, providing a path to identifying those changes that lead to changes in the molecular networks that define the biological processes carried out by the tissue.

**Figure 1:**
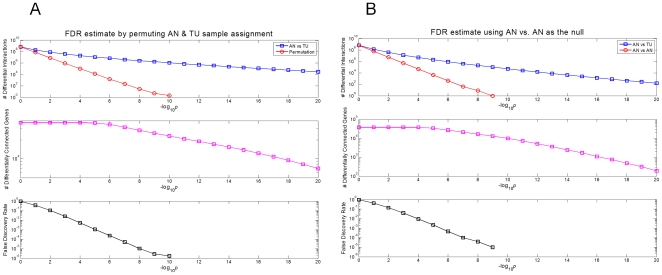
Genes differentially connected between AN and TU tissues. **A** Shown is numbers of the differentially correlated genes discovered using a meta-analysis procedure (Methods) between AN and TU tissues. For comparison the same analysis was run both in the real data and in a permutation of the AN and TU assignments. The top panel shows the number of differential gene pairs (Y-axis) for the real data (AN vs TU, in blue) in comparison to the permutation (red) as a function of the p value (shown as negative log10[P], on the X-axis). The number of differentially connected genes (middle panel) and false discovery rates (bottom panel) are also shown. **B** To establish that the differentially correlated gene pairs resulted from the difference between AN and TU tissue and not for example differences between individuals the same analysis was run using AN vs AN compared to AN vs TU using the same number of samples (Methods). The top panel shows the number of differential gene pairs (Y-axis) for both AN vs TU (blue) and AN vs AN (red) as a function of the p value (shown as negative log10[P], on the X-axis). The number of differentially connected genes (middle panel) and false discovery rates (bottom panel) are also shown.

To characterize changes between the AN and TU networks we used a meta-analysis procedure [19], [20] to test whether the magnitude of association between any given pair of genes in one tissue was significantly different from the association observed for that same pair in the second tissue (Methods). Significant correlation differences indicate the presence of connectivity differences between the AN and TU networks (Figure 1). At a Bonferroni adjusted, differential connectivity p = 7e-11 (<<1% family-wise error rate), we identified 1,156,638 differentially correlated pairs (only 2 would have been expected by chance), or roughly 12% of the 9,976,814 correlated gene pairs found in either the AN or TU tissues. We also empirically estimated the differential connectivity null distribution and observed no differentially connected pairs with p<7e-11 (Figure 1), suggesting that this degree of differential connectivity is highly significant.

To increase confidence that gene pairs identified as differentially correlated between the AN and TU networks reflected biologically relevant changes in network states related to tumorigenesis and tumor progression, we restricted attention to those differentially correlated gene pairs that were highly unlikely to have occurred by chance (p<1e-19, with a mean change in Spearman correlation coefficient between tissues of 0.73; no gene pairs observed in the permuted data, FDR<1e-6, see Figure 1). At this stringent cut-off, we identified 49,300 gene pairs covering 8,736 genes whose relationship differed significantly between the TU and AN tissues. Of the 49,300 differentially connected pairs identified, 42,179 (86%) were strongly correlated in the AN tissues, but significantly less correlated in the TU tissues, while only 7,121 pairs (14%) had stronger correlations in the TU versus AN samples, indicating that the network changes occurring in the tumor were more likely to destroy rather than create strong associations between expression traits.

**Figure 2:**
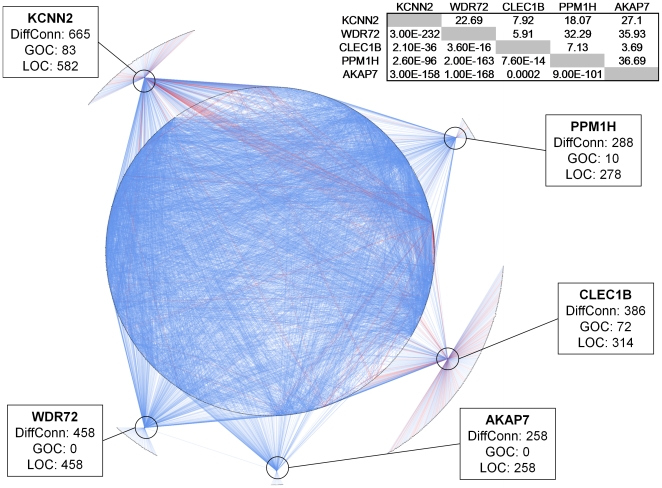
Differential correlations for the top 5 genes. The top 5 differentially connected genes and their differentially connected partners are shown. Each gene is represented by a blue oval and the top 5 are indicated by the boxes. For each of the top 5 genes also indicated is the number of differential correlations between AN and TU in total (DiffConn) and the numbers for gain (GOC) and loss (LOC) of correlation in each case. Lines connecting genes indicates that that pair was differentially correlated between AN and TU where both LOC (blue lines) and GOC (red lines) are indicated. Differential connections between the top 5 genes and any other gene are shown, as well as differential correlations between genes differentially connected to the top 5. The top 5 genes were found to be differentially correlated to a highly overlapping set of partners. Shown in the insert table (top right) is the Fishers Exact Test p value for overlap between differentially correlated gene partners for each of the top 5 genes (lower left of table). Also shown (upper right) is the fold enrichment for the overlaps relative to what would be expected by chance. As shown a complex web of differential correlations resulting from tumorigenesis is revealed.

**Figure 3:**
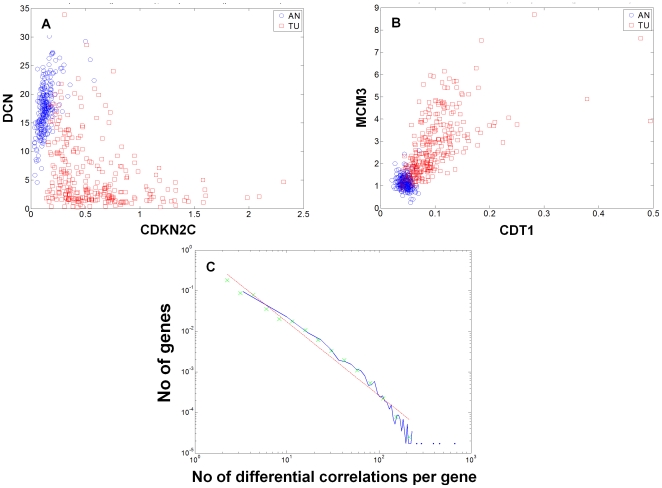
Differential correlations and connectivity structure between AN and TU tissues. To illustrate both loss and gain of correlation two cell cycle genes were chosen that have many differential connections: the cyclin dependent kinase inhibitor CDKN2C (**A**, 149 loss and 11 gains of connectivity) and the chromatin licensing and DNA replication factor CDT1 (**B**, 0 loss and 38 gains of connectivity). The expression intensity values for CDNK2C (X-axis) and DCN (Y-axis) are shown (**A**) in AN (blue, cc 0.548, p = 2e-20) and TU tissues (red, cc -0.358, p = 7.14e-10). The intensity values for CDT1 (X-axis) and MCM3 (Y-axis) are shown (**B**) in AN (blue, cc-0.153, p = 0.0166) and TU tissues (red, cc 0.673, p = 3.05e-38). Shown in **C** is the degree distribution for the 8,736 differentially connected genes as described in the text. The numbers of differential connections for each gene (log10, X-axis) was compared to the count (log10, Y-axis). As shown the distribution was scale-free, indicating that the identified genes tend to preferentially attach to a small number of hub genes in either tumor or adjacent normal tissues, but not in both.

To distinguish between the types of genes involved in differential connections we defined gain of connectivity (GOC) genes as those in which more than 90% of their differential interactions reflected correlations that were stronger in TU compared to AN. Similarly, we defined loss of connectivity (LOC) genes as those in which more than 90% of their differential interactions reflected correlations that were weaker in TU compared to AN (Supplementary Table S2). Although these are arbitrary cut-offs they serve to highlight the relative distribution of gain and loss of connectivity associated with tumorigenesis, given greater than 80% of the differentially connected genes fall in one of the two categories. Under this categorization there were 6,053 LOC genes and only 1,020 GOC genes. GO enrichment analysis after Bonferroni correction for the number of categories revealed that the GOC genes were enriched in cell cycle (3.93-fold enriched, p = 1.6e-20) and related processes (e.g., chromosome segregation, DNA replication, and spindle organization), while the LOC genes were mostly enriched for metabolic processes (1.11 fold enriched, p = 1.4e-20), especially those associated with mitochondria (1.56-fold enriched, p = 1.5e-20). These results suggest that the process of tumorigenesis is to a large degree one of disruption of normal networks (LOC), and to a lesser degree one of creation of new networks (GOC). Consistent with this, LOC events were enriched for normal liver function genes, where as the GOC events were enriched for genes involved in cancerous growth of the cell. GOC events, although smaller in number, may represent tumor specific functions required for disease progression and as such may be an interesting source of targets for HCC. To illustrate the nature of the differential connections the top 5 genes and the genes to which they were differentially connected are shown in Figure 2. Examples of individual gene pairs that were differentially connected in TU or AN tissues are shown in Figure 3, including the inhibitor of the G1 to S phase transition (CDKN2C with loss of connectivity) and DNA replication licensing factor (CDT1 showing gain of connectivity).

To assess whether the differential correlations were randomly distributed amongst the significant gene-gene correlations or whether there was some higher level structure, we examined the distribution of the number of differential correlations for each gene. We observed that whereas most genes participated in a small number of differential correlations, there was a subset of genes that participated in many differential correlations. In fact, the differential correlations closely followed a power law distribution that was quite different from what would be expected by chance (see Figure 3). This indicates that certain genes represent hub nodes in the differentially connected matrix that arose from tumorigenesis and as such may be of particular importance.

### sCNV explains a large fraction of TU expression variation

Given the large scale changes in expression and correlation structures arose during the process of tumorigenesis, we sought to identify the causal drivers of these changes. Somatic copy number variation is a common feature of many solid tumor types and has been associated with the aggressiveness of disease. For HCC in particular sCNV has been observed at the earliest stages of disease and increases in prevalence with disease progression [11]. We therefore assessed the prevalence of sCNV in HCC and to what extent it was associated with gene variation in the TU tissue.

**Figure 4:**
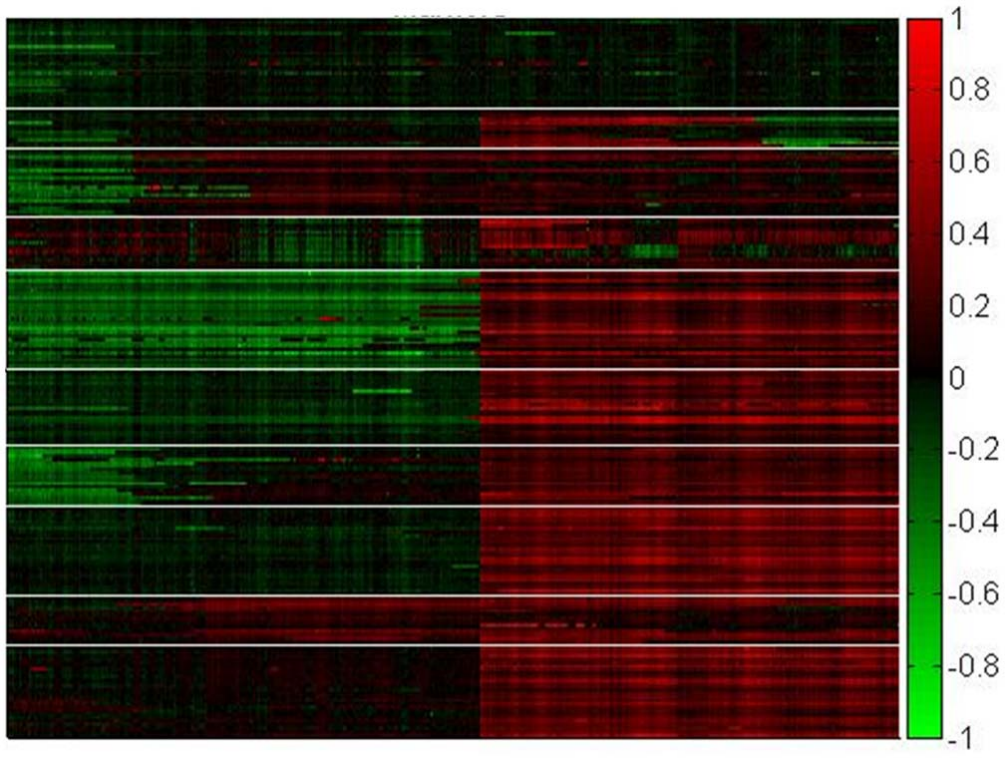
Copy number aberrations in HCC tumor samples on chromosome 1. Shown is a heat map of copy number aberrations (sCNV) for tumor derived samples (Y axis) clustered by K means into 10 groups, versus the linear positions through chromosome 1 (X axis). sCNV was estimated as described in methods and is indicated as a continuum of color from red (amplification) to black (no change) to green (deletion). A scale for the sCNV data is indicated on the right hand side of the heat-map (logR ratio from 1 to −1). As can be seen the majority of aberrations appeared to involve large chromosomal sections on the scale of whole chromosome arms.

DNA variation was assessed in the AN and TU samples using Illumina high-density SNP microarrays. sCNV were estimated using smoothed logR ratio's of adjacent markers at 32,711 evenly spaced loci through the genome (Methods). In the TU samples evidence of frequent amplification or deletion involving large genomic regions was seen (Figure 4). In contrast very few such events were observed in the AN samples with this analysis (4 AN samples were found to have limited evidence of copy number variation). sCNV variation was compared to gene variation in both the AN and TU samples.

**Figure 5:**
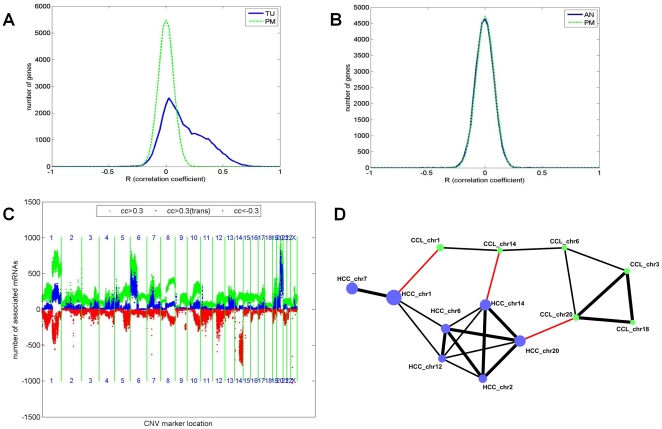
sCNV and expression correlations in cis and in trans. Distribution of correlations (X-axes) between genes (Y-axes) and the closest sCNV marker (in cis) in TU (**A**) and AN (**B**) tissues. The distribution of the real data (blue solid lines) was compared to permutation of the gene to marker connection (green dashed lines). No significant associations were seen in AN above what would be expected by chance. In TU there was a pronounced bias to positive correlations. **C**. Distribution of correlations between all genes (Y-axis) and all sCNV markers, shown linearly by chromosome location through the genome (X-axis). Chromosome boundaries are indicated by the vertical green lines and are numbered. Using a correlation cut-off of >0.3 (p<4.7e-4, FDR <0.02), the count of all genes (not including cis genes, green), positively correlated trans genes (red), or negatively correlated trans genes (blue) is indicated for each marker (trans here was defined as genes and sCNV markers falling on separate chromosomes). Several hotspots were apparent in which many genes were associated with sCNV at a particular locus, especially for regions on chromosomes 1, 2, 6, 7, 12, 14 and 20 (see text for additional discussion). **D**. Genes associated with sCNV hotspots in HCC and cell lines significantly overlap. Hotspot sCNV markers were selected by identifying regions associated with >500 genes (Pearson correlation coefficient>0.3) and then selecting the single top marker per chromosome. The genes associated with each hotspot marker were compared (blue circles, size equivalent to number of genes) and significant overlaps (Fishers Exact Test p<1e-6) are shown as edges connecting pairs of nodes. A similar analysis was performed on a collection of cancer cell lines (CCL, green circles). In this case a smaller number of total genes were measured (23,404 vs 37,585), so an equivalent fraction of the total genes (>370) significantly associated with a sCNV marker was required. The thickness of the edges connecting the nodes represents the enrichment of the overlapping genes in comparison to that expected by chance (observed overlap divided by expected; enrichment <5 fold – thin line, >5 – thick lines). As shown the genes associated with hotspots were shared within datasets. For example, the genes in HCC linked to the hotspots of chromosomes 2, 6, 12, 14 and 20 significantly overlap each other. Hotspot overlaps between CLL and HCC involving the same genomic regions are highlighted in red (chromosomes 1, 14 and 20).

Consistent with previous studies of other cancer types and radiation hybrids [4], [21], [22], [23], strong positive correlations between genes and sCNV markers were identified in cases where the corresponding genes overlapped or were near the sCNV marker being tested, referred to here as cis-acting associations (Figure 5A and Table S2). The most likely explanation for this observation in TU tissue is that sCNV induce proportional changes in genes that were proximal to the site of that sCNV. In contrast there were no cis-acting associations between AN CNV markers and AN genes beyond what would be expected by chance, indicating that the cis-correlations between sCNV and expression were tumor specific. Given this correlations to copy number variation were only investigated using TU tissue.

More generally, association tests between all TU genes and TU sCNV markers revealed many highly significant associations, referred to here as trans-acting associations (see Figure 5C). Several genomic loci were found to be associated with many more genes than would be expected by chance, referred to here as hotspot sCNV loci (see Figure 5C). We identified 7 hotspot sCNV loci on 7 different chromosomes that were each associated with >500 trans genes (correlation >0.3; FDR <0.001). The genes associated with these hotspots were highly overlapping, suggesting that sCNV at multiple loci coordinately drive networks of genes (Figure 5D and Table S3). To assess whether these hotspot loci were specific to HCC, we carried out a similar association analysis between sCNV markers and genes in a collection of ∼130 cancer cell lines (CCL) from multiple tissue types [24](Methods). Three of the 7 hotspot loci identified in the HCC data overlap sCNV hotspots in the CCL data. In all three cases the same genomic locations were involved. These data suggest that sCNV hotspots are not unique to HCC, but in fact may occur in many tumor types and can involve similar pairs of genomic loci and genes, perhaps driving core biological processes critical to tumor formation and progression. Consistent with this it has recently been reported that the structure of sCNV is frequently shared across multiple tumor types [25]. This might suggest that the cis and trans correlations reported here in HCC and cells in culture may be relevant to many tumors types with shared sCNV structure.

To establish the percentage of TU gene variation explained by any combination of sCNV markers, we constructed genetic models using a stepwise regression procedure for each gene (Methods and see Table S2). As a control we ran the same analysis using the permuted data where the connection between the genes and sCNV markers was randomized. Using the stringent cut-off of absolute correlation >0.3 (FDR <0.001), the amount of variance explained by sCNV was as high as 80%. Strikingly, greater than 40% of the genes represented on the microarrays used in this study (15,993 out of 37,585) were significantly associated with sCNV markers, where the average variance explained amongst these genes by the sCNV markers was 21.8%. For 3,031 of the genes (8.1% of the genes represented on the microarray) greater than 30% their variance was explained by sCNV markers (Table 1 & 2). Also, while cis-acting associations explained most of the sCNV associations, 6.6% of the genes (n = 2,490) had variance explained by sCNV markers that were other than cis, and a subset of genes (7.8%, n = 2,974) were found to have variance explained by more than one and up to five sCNV markers on different chromosomes. As shown above (see Figure 5) the majority of trans genes associate with a limited number of hotspots suggesting that variation at these limited number of loci was causing a significant proportion of the TU gene variation. These genetic effects on tumor gene expression are orders of magnitude larger than effects induced by germline DNA variation.

**Table 1:** Summary of regression analysis for all genes onto sCNV markers.

R2>cut-off	trans+cis	cis	pm trans+cis	pm cis
0.1225	14845	5427	1998	0
0.2	7169	2340	352	0
0.3	3031	759	100	0
0.4	878	205	31	0
0.5	195	40	5	0
0.6	28	1	1	0

Shown are the results of the regression analyses for each gene onto selected sCNV markers as decribed in Methods. **A.** Distribution of variance explained by sCNV across all genes. Counts for number of genes with various cut-offs for variance explained(“R2>cutoff” column 1) for all genes and all sCNV markers (“trans+cis”, column 2) and for cis markers only (“cis”, column 3) using the real data was compared to a similar analysis where the connection between the expression and sCNV markers was permuted (“pm_trans+cis” and “pm_cis”, columns 4 and 5).

**Table 2:** Distribution of number of genes by number of markers in model.

No markers	genes	pm
1	12592	2278
2	2974	202
3	395	25
>3	32	3

In addition the distribution of genes as a function of the number of markers included in the regression model is shown. The number of markers (“no markers”, column 1), was compared to the number of genes with that number of markers (“genes”, column 2) for the real data and identically processed permuted data (“pm”, column 3).

### Differentially correlated genes preferentially associate with sCNV markers in TU

To explore whether sCNV was driving coherent changes in networks that in turn induced phenotypic changes in the tumor, we tested for relationships between sCNV markers and differentially correlated genes between the AN and TU tissues. The differentially correlated genes were significantly enriched for genes associated with sCNV markers in cis (2.36-fold enriched; p<1e-300) as well as for the amount of variance explained (2.12-fold enriched, p<1e-300) and for the number of markers (1.89 fold enriched, p<1e-300) in the regression model. This enrichment held for GOC genes (cis 1.49 fold enriched, p = 1.03e-11, variance explained in regression model 1.55 fold enriched, p = 3.48e-25, number of markers 1.38 fold enriched, p = 1.04e-18) as well as LOC genes (cis 2.17-fold enriched, p<1e-300, variance explained in regression model 1.91-fold enriched, p = 1.1e-321, number of markers 1.81 fold enriched, p = 2.5e-287). The appearance of differential correlations in HCC therefore appeared to be largely explained by the effect of sCNV in TU tissue.

### Prediction of survival in AN and TU tissues

**Figure 6:**
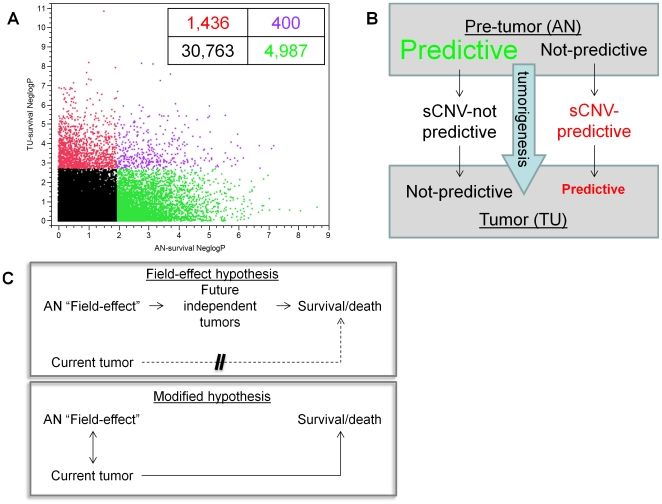
Relationship between genes in AN and TU and prediction of survival. **A**. Shown is the significance of association (as negative log10 of the Cox regression p value) found between all 37,585 genes in AN (X axis) and TU (Y axis) and survival. Genes found to be significantly associated with survival (FDR<0.1, Methods) are indicated in AN (green dots), TU (red dots) or both AN and TU (purple dots). As described in the text most genes predictive in one tissue were not predictive in the other. **B**. Shown is a representation of the network transformations associated with HCC tumorigenesis (transition from pre-tumor state, upper box, to the tumor state, lower box), where predictive genes in AN (green) largely lose their association to survival in TU following association to non-predictive sCNV. In contrast genes predictive of survival in TU (red) are largely not predictive in AN, and were preferentially associated with sCNV markers that were also predictive. Not shown are genes predictive in both AN and TU, and genes not predictive in either tissue. **C**. Shown diagrammatically is the “Field-effect” hypothesis as proposed [26] (upper box), where adjacent normal genes predict patient survival because they reflect a milieu (field-effect) in which future tumors are more or less likely to arise. In this model the current tumors do not have a large impact on outcome whereas future tumors do. A modification of this hypothesis is proposed here (lower box), where the adjacent normal genes represent a state that directly affected the probability of the current tumors arising and progressing. In this modified hypothesis survival or death is mediated by the current tumors. See text for additional discussion

We next characterized the relevance of the massive changes in gene networks to the clinical course of the disease by comparing the network changes to the subset of genes that predict survival. Genes predictive of survival were identified in AN and TU tissues using a Cox regression model (Methods, see Table S2). Approximately three times as many prognostic genes were found in AN (p<0.0112, n = 5,387; FDR <0.1) versus TU (p<0.002, n = 1,836; FDR <0.1). Although the predictive genes in AN and TU overlap more than would be expected by chance (1.52 fold enrichment, p = 6.8e-19, representing 7.4% and 21.8%, respectively, of the AN and TU predictive genes), there were many cases of genes highly predictive in one tissue but not in the other. For example, of the 5,387 genes predictive in AN, 4,987 (92.6%) were not predictive in TU, and of the 1,836 TU predictive genes, 1,436 (78.2%) were not predictive in AN, using the above cut-off criteria (see Figure 6A). In both cases our statistical power was 45% to detect genes predictive in one tissue that were identified as predictive in the other tissue (Methods), indicating that the minimal overlap was not a consequence of low statistical power.

### Genes predictive of outcome in AN and TU were enriched for differential correlations and association to sCNV markers

Using the AN genes predictive of survival (henceforth referred to as AN-survival genes), almost half (2,646 of 5,387, 49%) were found to be differentially correlated in the transition to tumor, which is 2.11-fold greater than would be expected by chance (p<1e-300). The AN-survival genes were also more likely to be correlated to sCNV in cis (1.36-fold enrichment, p = 1.38e-47), and to have a higher proportion of their variance explained by sCNV (1.32-fold enrichment, p = 3.94e-50) and by a larger number of sCNV markers (1.33-fold enrichment, p = 1.09e-53) in the regression model. Similarly genes predictive of survival in TU tissue (henceforth TU-survival genes) were enriched for the differentially correlated genes (1.33-fold enrichment, p = 3.5e-15), and for correlating to sCNV markers in cis (1.22 fold enrichment, p = 5.66e-8). TU-survival genes were not found to be significantly enriched for the total variance explained or the number of markers in the regression model.

The AN-survival genes reported here significantly overlap those previously reported for AN-tissue in HCC [26]. and this independent set was also enriched for differentially connected genes (1.79-fold enrichment, p = 1.7e-14), GOC genes (2.23-fold enrichment, p = 2.14e-9), and LOC genes (1.77-fold enrichment, p = 1.2e-11), demonstrating a degree of consistency among the observations in the two studies. Given the above analyses, it is clear that the large scale rearrangements associated with tumorigenesis in HCC were connected to clinical outcome.

If the transformation in predictive values was mediated by sCNV then, for example, AN-survival genes should be excluded from correlation to sCNV markers that were themselves predictive of survival (Table S4). This was in fact found to be the case after adjustment for the non-random distribution of gene:sCNV associations (fold enrichment 0.88, p<1e-300, Methods). In contrast genes that were predictive of survival in TU were enriched for associating with sCNV markers that were also predictive of survival (3.37 fold enrichment, p<1e-300). Therefore, this leads to a model (see Figure 6B) where sCNV in TU alters the expression of genes resulting in both differential correlations between genes as well as gain (TU-survival genes) or loss (AN-survival genes) of prediction of disease outcome.

### The probability of network transformation in HCC tumorigenesis was related to the pre-cancer state

Conceptually it is easy to understand genes predictive of survival in TU – these genes represent gene networks and associated functions in the tumor that were rate limiting for the future (from the time of surgery onwards) progression of the disease, where that progression involved continued evolution of the gene networks. In this scenario the probability of future evolution of the gene networks was predicted by the current state of the gene networks in TU tissue across the individuals at the time of surgery. In other words, the probability of future elimination of detrimental functions and assembly of beneficial functions for the tumor is predicted by certain genes in the current state and that is why they were predictive of survival.

Why genes in the AN tissue were predictive of survival and after the process of tumorigenesis were no longer predictive, is less clear. It has been proposed that AN-survival genes represent a so called “field-effect” and predict the likelihood of additional tumors arising and therefore survival [26]. If this were the case presumably the same field effect might have played a role in the appearance of the current tumors as well (see Figure 6C lower box). This modified hypothesis appears more parsimonious since invoking future tumors is not required (see Figure 6C). Consistent with this idea, our finding that the AN-survival genes were significantly enriched for genes that participate in the process of tumorigenesis (genes differentially correlated between AN and TU tissues), suggests that indeed a direct connection to the tumorigenesis that occurred in the tumors in the HCC cohort was present. The direct connection is further strengthened by the finding that the change in prediction of survival for genes from AN to TU tissue can be explained by association to sCNV markers as described above.

Despite the distance between the AN and TU tissues (at least 2 cm), the above direct connection between AN and TU tissues may indicate that either the tumor is influencing the AN tissue or vice versa, and it is this effect that explains the presence of AN-survival genes. If this were in fact the case then correlations between AN and TU derived gene variation should be present (variation of gene(s) in the tumor directly alters gene(s) in AN). Direct correlations between AN and TU genes is problematic because inter-individual variation would be expected to dominate. That is, a tumor may be most similar to the normal cells from which it was derived, but this does not represent AN to TU communication per se. Therefore, to focus on tumor specific gene variation we tested for the association of AN gene variation with TU derived sCNV markers, where the sCNV represents tumor specific gene variation. Although we cannot exclude more subtle effects, the number of associations found in the regression model was not different than those found with permuted data (FDR ∼1, see Table S5).

The absence of evidence indicating direct communication between AN and TU tissues leaves the possibility that the AN tissue retains to a significant degree the characteristics of the pre-tumor cells from which the tumor evolved. In this case, the variance of genes prior to tumorigenesis (as represented by non-cancer AN tissue) predicted the probability of tumorigenesis occurring, but after that process had occurred (in TU tissue) the same genes were no longer predictive (see Figure 6C lower box). Consistent with this, once the AN network was transformed by changes in gene-gene correlations driven by sCNV, the formerly predictive genes would no longer be predictive (see Figure 6B). Since the process of tumorigenesis is linked and perhaps driven by network transformation, genes predictive of that process were also predictive of survival.

We can derive from this hypothesis a testable prediction. If the starting state of the gene networks is a determinant of the likelihood of tumorigenesis occurring, then treatments that promote tumorigenesis should selectively alter genes that participate in the network transformation that characterizes that process.

To this end we took advantage of a genetic model of HCC where the oncogene MET was over-expressed in the livers of mice, resulting in a large increase in the numbers of HCC tumors for that strain [27]. The hypothesis above predicts that a treatment that promotes HCC tumorigenesis (MET overexpression), should, prior to the onset of tumorigenesis, selectively alter genes that participate in the network transformations associated with tumorigenesis. The microarray profiles of liver tissues derived from strains of mice that differed only in the presence (TRE-MET) or absence (LAP-tTA) of overexpression of the human MET oncogene at a time point prior to the appearance of tumors were generated and analyzed (Methods). The resulting pre-cancer liver MET signature (n = 3,873, ANOVA p<0.01, FDR<0.01, listed in Table S2) was found to be enriched for genes differentially connected in human HCC (1.27 fold enrichment, p = 2.9e-34). The MET signature was also enriched for the LOC genes (1.33 fold enrichment, p = 2e-31) and for genes associated with sCNV markers in cis (1.46 fold enrichment, p = 2.48e-70), variation explained by sCNV in the regression model (1.26 fold enriched, p = 3.62e-37), and the number of markers in that model (1.23 fold enriched, p = 1.03e-31). Further, the MET signature was enriched for genes predictive of survival in AN (1.24 fold enrichment, p = 1.5e-13) and to a lesser degree in TU (1.17 fold enrichment, p = 4.1e-3). Therefore, an alteration known to cause an increased probability of mouse HCC tumorigenesis (MET overexpression) altered genes that were enriched in genes that participate in the network rearrangements characteristic of human HCC tumorigenesis, enriched for genes associated with sCNV in the tumors and for genes predictive of survival in AN. This is consistent with the model that the starting state of AN-survival genes predicts the likelihood of tumorigenesis occurring.

### Enrichment of differentially correlated genes and GO terms in co-expression networks

We next constructed co-expression networks for the AN and TU tissues, using a method successfully applied to other large population based studies [13], [16], [28] (Methods), in order to place the core set of highly significantly differentially correlated genes between AN and TU in a more broadly defined biological context from which they came (AN) and to where they ended up (TU). Co-expression analysis in HCC AN and TU samples resulted in the identification of 25 subnetworks (modules) in AN tissue and 20 subnetworks (modules) in TU. For purposes of identification, the modules were named as colors in order of their size, where the prefix AN- and TU- indicated whether the module was specific to the normal or tumor tissue network, respectively (module membership listed in Table S2).

**Figure 7:**
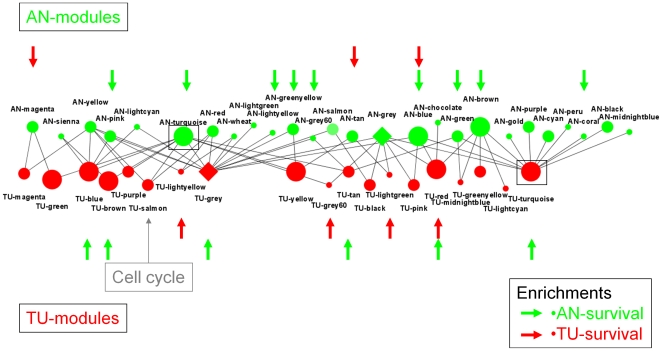
Overlaps between AN and TU derived co-expression modules. Co-expression modules were derived from both the AN and TU tissues (see Results and Methods) and are represented here as circles. Green (top) and red (bottom) circles are modules derived from AN and TU tissues respectively. The size of the circles indicates the number of genes in each including less than 100 (small), 100–500 (medium) and greater than 500 (large). Genes which were variant within the tissues but did not fall within a co-expression module are collectively represented by diamonds (one for each tissue). The largest modules from each tissue (AN-turquoise and TU-turquoise) are indicated by the boxes. Co-expression modules in AN (top) or TU (bottom) that were enriched for AN-survival genes (green arrows) or TU-survival genes (red arrows) are indicated. Additionally the concentration of cell cycle genes in the TU-salmon module is indicated.

A high level view of network rearrangements can be seen by comparing modules in AN and TU (see Figure 7 and Table S6). Many significant overlaps were seen indicating that the two tissues were far from randomly organized with respect to each other. Closer examination revealed support for the disruption and creation of co-expression networks found with gene-gene differential correlation data. For example, the genes in the largest module in AN (AN-turquoise) significantly overlapped the genes in eight different TU modules as well as genes that could not be placed in the TU network (referred to here as the TU-grey module), consistent with disruption of the AN-turquoise module. Similarly, genes in the largest module in TU (TU-turquoise), overlapped genes in 10 AN modules as well as genes in AN-grey, consistent with the creation of TU-turquoise as part of HCC tumorigenesis.

Consistent with the above interpretation of the AN and TU module overlaps was the finding that the differentially correlated genes were enriched in many of the modules. For example, 22 of the 25 AN modules and 13 of the 20 TU modules were enriched for genes containing differentially correlated genes, indicating that a majority of the subnetworks in the AN tissue representing many different biological functions (see below and Table S8) were disrupted as a result of the formation and progression of the tumor, resulting in a higher level re-organization (Table S7 A&B).

To assess possible biological functions represented by the networks, each module was tested for over-representation of genes from individual gene ontology categories. Given the large scale re-organizations between AN and TU, GO terms that were most significantly enriched for each module compared to all other modules were defined (Table S8). The purpose of this was to begin to define biological pathways that were uniquely disrupted in AN tissue and uniquely created in TU tissue (uniquely meaning that the enrichment was specific to a given module). Examples of this are the findings that components of the ribosome were uniquely enriched in the TU-grey60 module (“cytosolic ribosome”, 113.95 fold enrichment), and aspects of macrophage function were uniquely enriched in the AN-coral module (“regulation of macrophage differentiation”, 83.62 fold enrichment; see Table S8). In both cases these terms were not enriched in any other module of either tissue. Many unique or relative enrichments of GO terms were found for 14 of the 25 AN modules and 12 of the 21 TU modules representing a broad array of biological functions coherently represented in the networks and altered between tissues in HCC tumorigenesis.

### Towards the identification of TU specific functions, rate limiting for progression

Although there were many cases of GO terms uniquely enriched in TU modules, the functional significance of this remains to be determined. To this end enrichments of the AN-survival and TU-survival genes in all AN and TU modules was assessed (see Table S7 and Figure 7). The AN-survival genes were enriched in 9 of the 25 AN modules. Given the apparent independence of modules with respect to each other this suggests that multiple parallel networks must be altered in the tumor and as argued above, this is intimately linked to disease progression.

In the TU case 4 modules (TU-grey60: 9 fold enrichment, TU-lightgreen: 6.9 fold enrichment, TU-lightyellow: 5.6 fold enrichment and TU-red: 5.8 fold enrichment) were markedly enriched for TU-survival genes (see Table S7), suggesting they were important factors in disease progression. As judged by GO term enrichments these modules represent distinct biological functions including the ribosome (TU-grey60), aminoacyl-tRNA biosynthesis, ribosome biogenesis and the nucleolus (TU-lightyellow) and xenobiotic, amino acid and fatty acid metabolism, gluconeogenesis and mitochondrial proteins (TU-red).

Ribosome components have been clearly implicated in tumor initiation and progression in numerous cancer types [29]. In particular it has been argued that altered translation facilitates expression of many proliferation associated genes and may also regulate the endothelial to mesenchymal transition which is thought to be important in invasion and metastasis. The enrichment of TU-survival genes in TU-grey60 and TU-lightyellow suggests that translation and ribosome biogenesis were selected for alteration during tumorigenesis (both modules were enriched for genes correlated to sCNV hotspots, see Table S9), but remained rate limiting factors in progression. Particularly interesting is the finding of a protein complex involving DHX9, HNRPM, LSM2, HNRNPU and SNRPD1, 2 members of which (DHX9, HNRNPU) stabilize Myc mRNA [30] which was also a member of the TU-lightyellow module. Myc has also been shown to regulate ribosome biogenesis and translation and directly interacts with multiple ribosome components suggesting a feedback loop that may be important in cancer [31] and perhaps captured in the TU-grey60 and TU-lightyellow modules. Targeted interference in translation or ribosome biogenesis has been suggested as an efficacious therapy in other tumor types and should be explored as a therapy for HCC.

The TU-red module contains genes involved in a variety of metabolic functions many of which occur in the mitochondrion. Consistent with this a set of nuclear encoded mitochondrial genes [32] were enriched in the TU-red module (4.5 fold enrichment, p = 4.3e-41). Mitochondrial genes as a whole were enriched for becoming differentially correlated in TU (1.48 fold enrichment, p = 2.7e-30) and for becoming correlated to sCNV in cis (1.72 fold enrichment, p = 5.3e-62). Mitochondrial genes were also enriched in AN-survival genes (1.66 fold enrichment, p = 9.1e-24) likely indicating that network transformation involving this set was linked to disease progression. The mitochondrial set as a whole were not enriched for TU-survival genes, but perhaps significantly those present in the TU-red module were unusual in that they were predictive both in AN and TU tissues (16.89 fold enrichment of genes predictive of survival in both AN and TU, p = 3.5e-87). This suggests that formation of the TU-red module was important at both early and later stages of tumor development, and that this is centered on a subset of mitochondrial genes.

It has been reported that a high fat diet in a genetically susceptible background induces HCC in mouse [33], and that obesity and metabolic disorders are risk factors for HCC susceptibility and prognosis [12], [34]. Interestingly the signature of a high fat diet in mouse liver was enriched in the TU-red module (2.88 fold enrichment, p = 5.84e-12) linking environmental risk factors with a molecular network predictive of survival and biologically relevant to metabolic disease.

## Discussion

Copy number aberrations are widely observed in solid tumors and are likely the result of altered fidelity of DNA replication, repair, checkpoints and/or chromosome segregation. These processes leading to sCNV are by their nature intrinsic features of cancer cells and occur in an undirected manner in terms of chromosome location and direction of change (amplification versus deletion). Since generation of sCNV is ongoing and will lead to neutral, increased, or decreased fitness of the corresponding cell in its environment, different derivatives will have consequently different abilities to grow and survive, therefore leading to evolution of the tumor over time [6]. Clinically relevant tumor samples are therefore likely to represent the cumulative result of undirected generation of variance (random) followed by selection (non-random).

**Figure 8:**
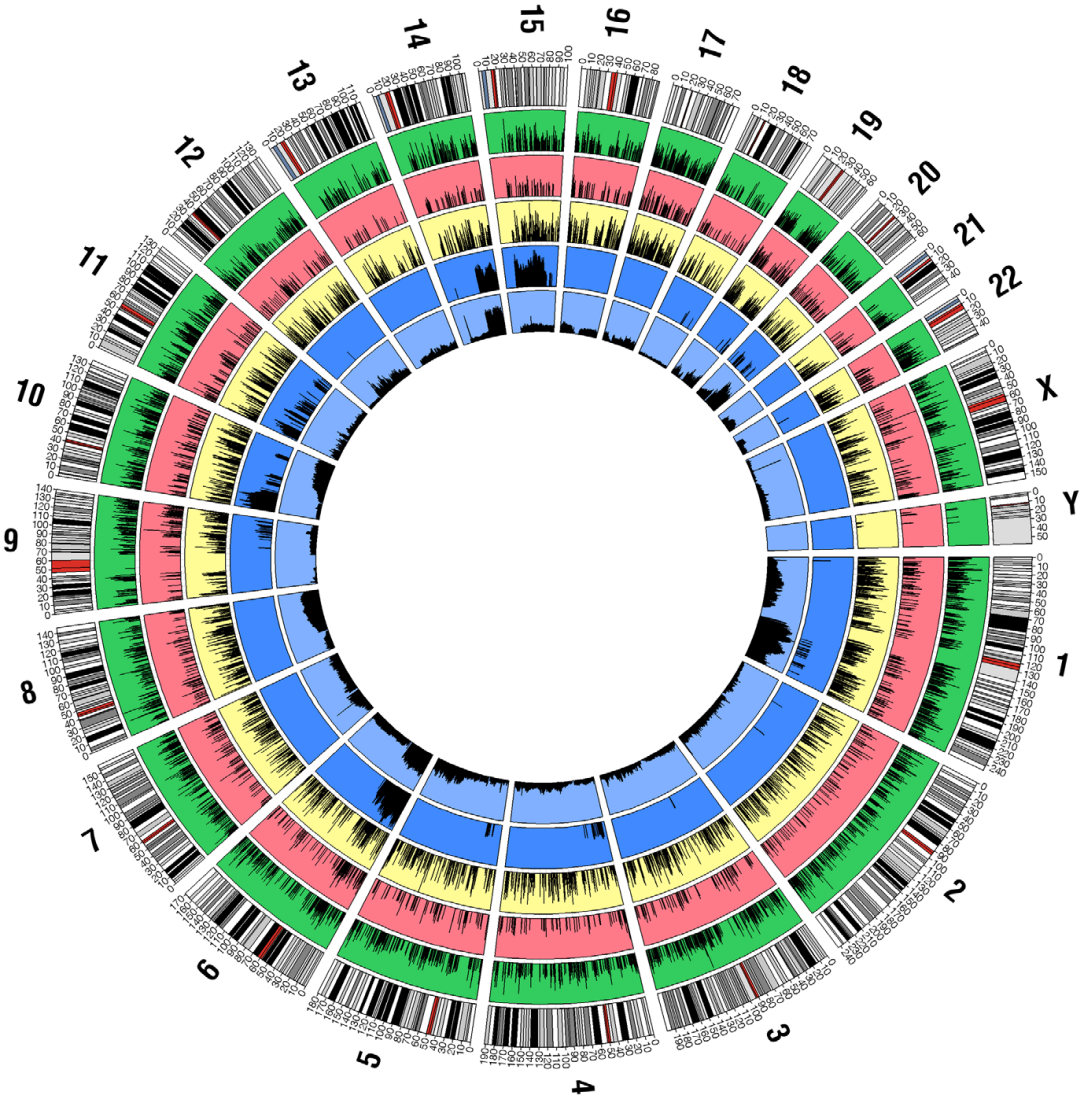
Global distribution of survival associated genes and sCNV, differentially connected genes and gene-sCNV correlations. Shown is a high level summary of various relationships described in this work. The graph was assembled using the Circos software (http://mkweb.bcgsc.ca/circos/) and data in Tables S2 and S4. Genomic positions are indicated on the outer circle with both chromosome (large numbers) and nucleotide position (smaller numbers, x1,000,000 nt) as indicated. The bars on the outer circle indicate the positions of cytobands. The 2^nd^ (green background) and 3^rd^ (red background) circles indicate the genes (by chromosomal location) that predict survival in AN or TU tissues respectively where a longer line indicates greater significance (see Table S2 and text). The 4^th^ circle (yellow background) indicates genes found to be differentially correlated between AN and TU tissue where the length of the line is proportional to the log10 of the number of differential correlations for that gene (see Table S2 and text). The 5^th^ circle (dark blue background) shows sCNV markers that predict patient survival where a longer bar is more significant (see Table S4 and text). The 6^th^ and innermost circle (light blue background) shows the number of genes correlated to each sCNV marker (cis and trans correlations, see Table S4 and text).

Using HCC and adjacent normal liver samples we investigated the gene and sCNV changes associated with tumorigenesis by comprehensively discovering the significant relationships within and between DNA copy number variation, global gene expression in TU and AN tissue and patient survival (See Figure 8 for global summary). Analysis of these data revealed the appearance of highly significant network changes as shown by gene pairs differentially correlated between AN and TU tissue. Interestingly this process largely consisted of loss of correlation in the TU samples consistent with disruption of normal networks. A subset of the changes observed involved gain of correlation in TU indicating the formation of new networks in some cases. Consistent with the view that loss of connectivity may represent loss of functionality and gain of connectivity may be gain of functionality, the LOC subset of genes was enriched for genes involved in normal liver function that might be expected to be largely extraneous to the needs of the tumor, whereas the GOC subset of genes is enriched in the essential tumor function of cell cycle. This appearance of loss and gain of connectivity during tumorigenesis may therefore be analogous to the long established concepts of tumor suppressers and oncogenes. Given the relative abundance of LOC versus GOC events this implies that tumorigenesis in HCC at least is to a large degree one of disruption of tumor suppressing normal networks. Although smaller in number the GOC genes likely represent functions selected as important for disease progression and as such may be important points of intervention (see also below).

Genes in TU were found to be strongly associated in cis and in trans with sCNV frequently involving large chromosomal regions. Within the architecture of sCNV-to-gene associations a number of hotspots were found where many more genes were associated with a particular marker than would be expected by chance. Additionally the genes associated with the hotspots were highly overlapping suggesting that multiple different loci may coordinately regulate a core subset of genes. The finding of hotspots in cancer data may not be unique to HCC in that similar associations, even involving the same genes and sCNV loci were found in an independent collection of cancer cell lines. Given the common architecture of sCNV across many tumor types [25], the cis and trans correlations documented here may therefore be relevant to a broad range of diseases. The differentially connected genes between AN and TU tissue were also significantly enriched for association to sCNV markers in TU suggesting that the network transitions and associated functional changes may be mediated by somatic sCNV.

A surprising finding in this study was that at the same FDR, three times as many genes predictive of survival were found in AN than in TU tissue. Furthermore, although the AN-survival and TU-survival genes overlapped more than would be expected by chance, the majority of genes in each case were not predictive in the other tissue. A direct connection between the altered predictive value of the genes in AN and TU was found by association to sCNV markers where AN-survival genes were preferentially associated with sCNV markers in TU that were not predictive, and TU-survival genes were enriched for association to predictive sCNV markers. It therefore seems that the sCNV in tumors may be sufficient to explain the transformation of the predictive value of genes in AN versus TU.

To directly address the hypothesis of whether the pre-existing state of genes that participate in network rearrangements are a determinant of the probability of that transformation occurring, we measured the transcriptional signature of a treatment that promotes HCC tumorigenesis. Human MET overexpression in mouse liver produced a gene signature, prior to the appearance of tumors, that was significantly enriched in the human AN-survival genes, in genes that participate in human HCC network changes, and in genes associated in human HCC with sCNV. This is directly supports the hypothesis that the pre-tumor state, as measured by the AN tissue, was a significant determinant of the large scale network transformations required to produce HCC tumorigenesis.

There are a number of interesting ideas that derive from this hypothesis. One is that MET overexpression causes increased tumorigenesis by altering the genes (networks) that participate in that transition, or in other words the starting state of these genes is causally related to the probability of the future transformation occurring. Similarly then, the starting state of the AN-survival genes may be causal for the probability of network transformations involving them in human HCC. The TU-survival genes in an analogous manner may also be causally related to the probability of future network evolutions relevant to disease progression. This further suggests, that as in the MET case, manipulation of the relevant genes will alter the probability of HCC network changes and tumor evolution occurring.

The finding that AN-survival genes for the most part lose their predictive value in tumor is interesting. By implication once the network transformation has occurred those genes and their associated functions were generally no longer rate limiting. This is apparently largely true for both disruption of normal networks (LOC genes) and creation of new networks (GOC genes). From a perspective of targeting tumors, disruption of normal networks may be hard to reverse in practice. However the creation of new networks may represent functions that the tumor has gained or emphasized relative to the tissue from which it was derived. As such these functions may make desirable targets in that the tumors have selected for them and the selection process may relate to survival. Targeting these new networks may therefore disrupt essential tumor specific functions.

Finally, TU-survival genes may also represent an opportunity for intervention in that as described above they may causally relate to the probability of future disease progression. Investigation of co-expression networks highlighted 4 co-expression modules that were enriched for TU-survival genes. Two of the 4 modules were strongly linked to ribosomes and ribosome biogenesis, which have been linked to aggressive disease in other tumor types and individual components when either over or under expressed promote tumorigenesis. Myc has been shown to alter a number of ribosome components and in turn can be regulated by them and was found here to be in the same co-expression network. This suggests that altered translation maybe a significant factor in HCC disease progression. A third co-expression module was unusually found to be enriched for both AN and TU-survival genes and centered around metabolism and the mitochondrion. Although it is speculation it is tempting to suggest that this group of genes may represent the molecular equivalent of the epidemiological observation that obesity is a risk factor for susceptibility to HCC and survival after diagnosis. Interestingly it was recently suggested that switching to a low fat diet alters the course of disease in mouse models.

## Materials and Methods

### Patient demographic and clinical parameters

Demographic and pathologic parameters for the 272 ethnic Chinese HCC patients who received curative surgery and used in this study are shown (see Table S1, see also [17]). Half of the patients (51.1%) suffered from tumor recurrence during the follow-up period. The primary endpoints measured were overall survival, disease-free survival (DFS), and tumor stage (pTNM [35]). In a simple Cox model, the endpoints were found to be significantly associated with tumor size, serum alpha fetoprotein (AFP) levels, total albumin concentration (ALBU), venous infiltration (veninv), AJCC stage [35], and the number of tumor nodules (NOTN). Patient and ethics approval for this study was obtained from Institutional Review Board of the University of Hong Kong/Hospital Authority Hong Kong West Cluster (HKU/HA HKW IRB).

### Sample processing to isolate DNA and RNA

Tissue milling and division was accomplished as follows. Flash frozen tissue was placed in a chilled milling tube along with a stainless steel bead, dipped in a liquid nitrogen bath and loaded onto the QIAGEN TissueLyser for milling (30 Hz in 30 second intervals). Multiple cycles of milling were sometimes required to achieve complete pulverization of the tissue to a fine powder. After milling, the tissue powder was recovered and rapidly manually split for extraction of DNA and RNA. At all times sub-zero temperatures were maintained.

Homogenous tissue aliquots resulting from the milling process were digested overnight with proteinase K. A portion of the digest was then dedicated to DNA extraction using an automated protocol based on the Agencourt (Beverly, MA) Genfind SPRI® (Solid Phase Reversible Immobilization) paramagnetic bead-based technology for the selective immobilization of nucleic acids onto magnetic microparticles. Following the precipitation of the eluted DNA, samples were quantitated using PicoGreen (Invitrogen) and qualified by agarose gel electrophoresis.

Isolation of RNA was achieved using the following procedures. The milled tissue samples were homogenized in cryopreservation tubes with a vortex mixer after addition of 750 to 1000 uL of 100% TRIzol. 100% Chloroform was added to the TRIzol/GITC lysate (1∶5 ratio) to facilitate separation of the organic and aqueous components using the phaselock (Eppendorf) system. The aqueous supernatant was further purified using the Promega SV-96 total RNA kit, incorporating a DNase treatment during the procedure. Isolated total RNA samples were then assayed for quality (Agilent Bioanalyzer) and yield (Ribogreen) metrics prior to amplification.

### RNA amplification and hybridization

Samples were amplified and labeled using a custom automated version of the RT/IVT protocol and reagents provided by Affymetrix. Hybridization, labeling and scanning were completed following the manufacturer's recommendations (Affymetrix). Sample amplification, labeling, and microarray processing were performed by the Rosetta Inpharmatics Gene Expression Laboratory in Seattle, WA.

### Gene expression data processing

The intensity of all gene array experiments were normalized together using the RMA method [36]. Afterwards, the intensity was adjusted for gender and age of the patients. To avoid the influence of outliers, we fitted the robust linear model (rlm, M-estimation with Tukey's bisquare weights, implemented in R statistical package), and used the residuals as the gene trait in all subsequent analyses. In brief, for every gene (for example, gene_j_) we fitted a linear model:
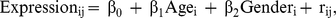
where Expression_ij_ is the expression level for gene_j_ in the i^th^ patient; and Age_i_ and Gender_i_ are age and gender of the i^th^ patient. Each patient contributes to the model differently according to his/her Tukey's bisquare weights derived from r_ij_.

The expression data has been deposited in GEO (GSE25142, http://www.ncbi.nlm.nih.gov/geo/).

### DNA genotyping

Whole genome genotyping was performed using the Illumina Infinium Assay, following all manufacturer specifications. Approximately 750 ng of high molecular weight genomic DNA was used for each sample to produce genotyping calls across 650,000 SNPs distributed throughout the human genome. The technology used was reported [37], and current protocols are available from www.illumina.com. To identify copy number variation, we obtained the log R ratios and smoothed the data using a 40-SNP window with a 20-SNP step to minimise noise and maximise signal. The genotyping data has been deposited in GEO (GSE28127, http://www.ncbi.nlm.nih.gov/geo/).

### Enrichments tests for gene sets versus Gene Ontology (GO) and other terms

The gene sets as defined in the text were compared to independently derived collections of genes representing biological functions in various public and other databases using the Fisher Exact Test. The p values in all cases represent the significance of the Fisher Exact Test statistic under the null hypothesis that the frequency of the indicated gene set is the same between the network module and the reference set of genes taken to be the set of genes comprising the network of interest. Given many such tests were performed over many gene sets, a conservative Bonferroni adjustment for multiple testing was employed by multiplying the resultant p values by the number of tests performed.

For the identification of GO terms uniquely or relatively over-represented in co-expression modules the following procedure was used. As above enrichments of GO term for each co-expression module was assessed using Fishers Exact test and corrected for multiple testing by multiplying the p values by the number of tests. An adjusted p<0.05 was used as a cut-off. Fold enrichments (observed overlap divided by expected), were then used to compare enrichments of individual GO terms between modules. What is shown in Table S8 are cases where a GO term was more highly enriched (fold enrichment) in comparison to all other modules.

### Meta-analysis of gene-gene correlation

For each gene pair *(i*,*j)* and their Spearman correlation coefficients, *r_tij_*, where *t =  {tumor*,*normal}* is measured in tumor and adjacent normal tissues, respectively, we first transformed the correlation coefficients into Fisher's Z-statistics:
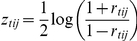
which follows a normal distribution with mean zero and standard deviation of 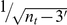, where *n_t_* is sample size. A heterogeneity statistics, *Q*, is then computed as follows
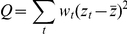
where *w_t_ = n_t_−3* and 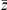 is the average *z* between tumor and adjacent normal tissues. Under the homogeneity assumption, the *Q* statistics should follow a *x^2^* distribution with one degree of freedom under the homogeneity assumption. The larger the *Q* statistic is, the less similar the two gene's relationship is between the two tissues. We chose *Q_0_ = *80, corresponding to a p-value of *p_0_ = *1
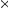
10^−20^, as the cutoff to determine the statistical significance of the heterogeneity *Q* statistic. Gene pairs with *Q>Q_0_* were considered as significantly differentially connected and were retained for subsequent analysis. We further estimated that the false discovery rate at our selected cutoff was *FDR <*1
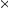
10^−6^ by repeating the same meta-analysis procedure on a permuted version of the original data in which the sample labels were randomly assigned.

### DNA/gene associations and permutations

For DNA/mRNA associations, smoothed logR ratio's were derived by taking the average intensity for 40 adjacent markers sequentially through the genome with a step size of 20 markers. This procedure results in 32,711 sCNV markers evenly distributed through the genome. The degree of DNA association to mRNA was measured by using Pearson correlation coefficient on the smoothed log R ratio data in a pair-wise fashion, using gene expression, adjusted by gender and age as described above. *Cis* effects were defined as the closest DNA marker within 5 Mb of the gene encoding each mRNA. The significance of DNA/mRNA correlations, was assessed by permuting the sample assignments such that the connection between the two data types is destroyed but the connections with each type (DNA/DNA and gene/gene) are maintained.

The smoothed logR ratio procedure was compared to a published segmentation method [38] which is reported to be comparable to other published methods [39], by assessing the number of significant associations between copy number variation (as defined by each method) and expression variation in cis for a given FDR. In a head to head comparison the smoothed logR ratio method discovered larger numbers of cis associations at all FDR cut-off's in comparison to the published method (see Table S10). In light of this the smoothed logR ratio method was used for all estimations of copy number variation.

As described in the text certain loci are associated with hundreds of genes. Using an arbitrary cut-off for markers associated with >500 genes (correlation >0.3), 7 loci were identified as described in the text. To assess if these “hotspots” could have occurred by chance the connection between the gene expression and sCNV data was permuted while maintaining the internal structures of expression and DNA variation. After 10 such permutations no hotspots were discovered using the criteria above, therefore we concluded that the observed hotspots were not likely to have occurred by chance.

### Stepwise regression to quantify sCNV contribution to mRNA variation

A forward stepwise multi-linear regression model was used to derive the adjusted squared regression coefficient (r2) for the proportion of mRNA variance accounted for by sCNV at multiple loci. For a given mRNA, we first fitted the initial model with the most correlated sCNV marker (p value cut-off  = 5.1e-6, 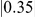), and then added another predictor from a different chromosome and compared the RMSE (mean squared error) of the new model to the previous one. If the new model had a smaller RMSE (mean squared error), then it was added to the model. This was repeated until no further improvement for the model was seen.

### Identifying expression traits and sCNV markers significantly associated with clinical endpoints

To explore the connections between sCNV markers, expression variation and disease endpoints, we identified those traits detected as most highly associated with survival in AN or TU tissue using a simple Cox regression model. In all cases a false discovery rate of 0.1 was derived from 100 permutations of the data. This resulted in 1,836 genes (p<0.002) and 3,223 sCNV markers (p<0.0089) in TU and 5,387 genes (p<0.0112) in AN tissue associated with survival.

### Enrichment of AN and TU-survival genes for association to sCNV markers

To adjust for the non-random distribution of significant associations between genes and sCNV markers through the genome, the following adjustment was made. For each sCNV marker the expected number of associations was derived by multiplying the test gene set (AN or TU-survival genes) by the total number of genes associated with that marker for a given cut-off and then dividing by the total number of genes measured (n = 37,585). This expected value was then used to divide the observed number of genes for the test gene set for the sCNV marker in question (i.e.observed divided by expected). The resulting ratio's were then used to assess the relative enrichment of AN and TU-survival genes for association to sCNV markers that were or were not predictive of survival as defined above.

### Description and analysis of cancer cell line (CCL) data

#### CCL cohort description

131 cancer cell lines from Caucasian donors were used in this study which has been more fully described elsewhere [24]. These cell lines were derived from various tumor tissues (31 colon, 26 leukemia/lymphoma, 20 lung, 15 skin, 6 breast, 6 bladder, 5 pancreas, and 22 from other tissues like stomach, brain, prostate, cervix, bone, liver, tongue, and uterus).

#### CCL Array CGH data

The Parallele MIP (Molecular Inversion Probe) platform [40] was selected to identify copy number variations in these cell lines. Approximately 8 ug of genomic DNA from each of the human tumor cell lines was extracted from cultured cell lines. A total of 17,000 SNP markers were genotyped using the MIP platform. Derivation and normalization of copy number for each markers was performed exactly as described [40]. To reduce the noise, copy number data for each marker was smoothed using adjacent markers (markers located 300 kb up or down-stream) [40].

#### CCL mRNA Expression data

Labeled cRNAs from mRNA samples obtained from the cell lines were fragmented to an average size of approximately 50 to 100 nucleotides then hybridized to Agilent Human 3.0 A1 arrays. Fluorescence intensities of the scanned images were quantified, normalized, and balanced and then gene expression (mlratio) was measured relative to a common reference RNA pool (Human Universal Reference RNA, Stratagene, La Jolla, CA), by using Rosetta Resolver Agilent Error Model. This data has been loaded into NCBI GEO database, with accession number GSE13598: (http://www.ncbi.nlm.nih.gov/geo/query/acc.cgi?acc=GSE13598).

#### CCL Tissue/cancer type effect on sCNV

To assess the presence of tissue/cancer type effect on sCNV, we first assigned each cell line to one of the eight tissue types of origin based on available annotations, then ran a one-way ANOVA analysis on every marker. Permutations were done by randomizing the tissue assignment. Finally the original sCNV markers were adjusted by tissue means to normalize the tissue effect. The same process was used to adjust mRNA expression data.

#### CCL DNA/mRNA associations and permutations

The degree of DNA/mRNA association was measured by using Pearson Correlation Coefficient on log2 transformed, tissue-adjusted copy number data in a pair-wise fashion. For *cis*-acting effect, we matched mRNA's probes on the expression arrays with the closest DNA copy number marker within 5 Mb. To assess the significance of DNA/mRNA correlations, we did the same analysis on permuted datasets, in which the links between sCNV and their original cell lines were randomly shuffled. Since this study focused on relationship among unlinked sCNV, when permuting the DNA copy number data, we preserved intra-chromosomal marker correlations.

### MET mouse liver signatures

The mice used in this study have been described [27]. All mice were in the FVB genetic background. The mice overexpressing human MET carried one copy of the LAP-tTa transgene (the liver-specific LAP promoter driving the Tet-VP16 transactivator) and one copy of the Tre-Met transgene (Tet-operator regulated human MET gene). The presence of both transgenes in these mice results in expression of the human MET gene specifically in and throughout the liver (referred to henceforth as the TRE-MET strain). A strain carrying two copies of the LAP-tTa transgene only was used as a control (referred to henceforth as the LAP-tTA strain). Seven mice of each of the two strains were sacrificed at 6 or 7 weeks of age prior to the appearance of tumors and the livers collected and processed for gene expression profiling (as described above) at the Rosetta Gene Expression Laboratory. As a control for the ability of the MET transgene to produce tumors, a parallel set of 7 mice for each of the two strains was sacrificed after 14 weeks, where 2 or more tumors per liver were found in each of the MET overexpressing mice and none in the control strain. MET specific signatures were defined as genes with an ANOVA p<0.01 between the LAP-tTA and TRE-MET derived samples at 6-7 weeks of age.

### Reconstruction of tumor and adjacent normal coexpression networks

A previously described weighted gene coexpression network reconstruction algorithm was employed to reconstruct the coexpression networks [13], [16], [28]. The weighted network reconstruction algorithm involved first constructing a matrix of Pearson correlations between all gene expression pairs. The correlation matrix was then transformed into an adjacency matrix using the power function *f (x) = x^β^*. The adjacency matrix defines the weighted coexpression network. The parameter *β* of the power function was determined such that the resulting adjacency matrix was approximately scale-free. To measure how well a network satisfies the scale-free topology property, we used the model fitting index proposed by Zhang & Horvath. This index is defined as the coefficient of determination (i.e., *R^2^*) of the linear model constructed by regressing Log(*p(k))* onto log(*k*), where *k* represents the degree of a given node (i.e., the number of edges connecting to the given node), and *p(k)* is the frequency distribution of the degree *k* in the coexpression network. The model fitting index of a perfect scale-free network is 1. The exponent of the power function, *β*, was chosen to be the smallest value such that the coexpression network exhibited the scale free property (*β* = 3.5 in this case). The degree distribution of the coexpression network approximates a power law (*p(k)∼k^-1.36^ p(k)∼k^-1.93^*), with a model fitting index >0.7.

## Supporting Information

Table S1 **Demographic and clinical characteristics of HCC patients.**
(XLS)

Table S2 **Summary by gene.** Shown are a number of measures (columns) derived from this analysis for each gene (rows). The columns are as follows “idx” is a unique row identifier, “Reporter Id” indicates the probe on the array; “chr”, “pos” and “cytoband” refer to the gene location by chromosome nucleotide position and cytoband respectively; “Symbol” indicates the gene name; “Entrez” indicates the Entrez Id; “transcript” lists commonly used transcript identifiers for each probe; “AN-module” and “TU-module” indicate membership in co-expression modules in the AN and TU tissue respectively; “Pval_TUvsAN” is the paired t-test p value for the difference in expression between AN and TU tissues; “Connectivity” indicates the total number of differential connections (gain plus loss) as defined in the text, and the number of gain ("#GOC") or loss ("#LOC") of correlations in tumor; “cis_CC” indicates the Pearson correlation coefficient between each gene and the closest CNV marker (in cis); “adjusted_r2” indicates the variance explained for each probe using the linear regression model as described in the text and methods; “N_Markers_in_Model” is the number of CNV markers used in the linear regression model where each marker is from a different chromosome (see text and Methods); “Pval-AN-survival” and “Pval-TU-survival” indicates the Cox-regression p value for prediction of survival using either AN or TU expression values respectively; “Sig-AN-survival” and “Sig-TU-survival” indicate genes significantly predictive of survival (FDR<0.1), where “1” =  predictive and “0” =  not predictive for AN and TU tissue; “MET-Sig” indicates genes significantly altered by MET expression in mouse liver (“1”) or not significantly altered (“0”). Please refer to the text and Methods for fuller descriptions of the various measures.
(XLS)

Table S3 **Overlaps between genes correlated to sCNV hotspots.** Overlaps between genes correlated to the top 7 sCNV hotspots (see Results and Methods) were tested. Hotspots are identified by the chromosome on which they resided (top row and first column). The Fisher Exact test p value for the overlap is shown for each comparison below and to the left of the grey boxes. The fold enrichment over chance (observed divided by expected) is shown above and to the right of the grey boxes. Fold enrichments are not shown for non-significant overlaps.
(XLS)

Table S4 **Relationship of sCNV markers to survival.** Listed are the relationships found between sCNV markers genome wide and survival using Cox regression (Methods). “Idx” indicates a unique identifier for each sCNV marker, “SNPid” is the SNP identifier for the SNP at the center of the smoothed window of adjacent sCNV markers (see Methods for derivation of smoothed sCNV markers), “chr” indicates the chromosome, “pos” is the nucleotide position of the middle of the smoothed with of markers, “CNV2GE_correlation” indicates the number of genes significantly correlated to each sCNV marker, “NeglogP-CNV-Survival” shows the negative log10 of the p value for prediction of survival using Cox regression, and “Sig-CNV-Survival” indicates markers considered to significantly predict survival where “1” is significant and “0” is not significant.
(XLS)

Table S5 **Summary of linear regression analysis for AN expression versus TU sCNV markers.** The distribution of variance explained by sCNV markers in the regression models for all genes is shown (Methods) using AN expression and TU derived sCNV markers. Counts for number of genes with various cut-offs for variance explained (“R2>cutoff” column 1) for all AN genes and all TU sCNV markers (“trans+cis”, column 2) and for cis markers only (“cis”, column 3) using the real data was compared to a similar analysis where the connection between the expression and sCNV markers was permuted (“pm_trans+cis” and “pm_cis”, columns 4 and 5). As shown the real data was not significantly different that the permuted data indicating a lack of detectable signal.
(XLS)

Table S6 **Co-expression module to module overlaps between tissues.** Shown are the overlaps found between co-expression modules derived from AN and TU tissues. “Set1” and “Set2” indicates the AN and TU modules tested respectively. “Pval” and “Fold” indicate the Fishers Exact test p value for enrichment of overlap and the fold increase in the overlap versus what would be expected by chance respectively. Results with a p value <1e-3 are shown.
(XLS)

Table S7 **Enrichments of predictive and differentially correlated genes in the AN and TU co-expression modules.** **A** Co-expression modules tissue, name and number of genes in each module (columns 1–3) are indicated. The number of GO terms enriched, and the top GO terms enriched for each module with enrichment p value and fold enrichment are also shown (columns 4–7) and the total number of GO terms enriched. Enrichments of genes predictive of survival in AN and TU tissue (columns 8–10 and 11–13, respectively), and for genes differentially correlated (columns 14–16), and the gain (columns 17–19) and loss (columns 20–22) are also shown. All p values were from the Fishers Exact test and fold enrichment were calculated by dividing the observed overlap by the expected overlap. **B** is as for **A** but with modules derived from TU tissue.
(XLS)

Table S8 **GO term enrichments for AN and TU co-expression modules.** Shown are the top GO term enrichments as judged by the relative fold enrichment for each AN and TU co-expression module. “Similar Set” indicates the GO term, “Module” indicates the co-expression module. “Fold” indicates the fold enrichment of the overlap versus expected, and “Diff” indicates the difference in fold enrichment between the indicated module and the next most enriched module from either AN or TU tissue. In cases where “Fold” and “Diff” are the same, there were no other significant enrichments for that term in any other module. That table is ranked in descending order by the fold enrichment.
(XLS)

Table S9 **Overlaps between sCNV hotspot correlated genes and co-expression modules.** Shown are the overlaps found between genes correlated to the top sCNV hotspots (“Hotspot”) and co-expression modules from AN and TU tissue (“Module”). Also indicated is the fold enrichment of the overlap versus the random expectation in each case (Fold). Only enrichments with a Fishers Exact test p<1e-3 are shown.
(XLS)

Table S10 **Comparison of CGHseq and smoothed logR ratio methods of estimating sCNV.** Shown is a comparison of methods for estimating copy number aberrations as described in the Methods. The ability of each method to detect associations between DNA variation and gene variation in cis for a given FDR was assessed. Shown are the FDR cut-offs applied (“FDR”, column 1), and the number of significant associations between genes and copy number aberration in cis using the CGHseg method (columns 2 and 3) and the smoothed logR ratio method (column4 and 5) for the real data (columns 2 and 4) and permuted data (used to calculate the FDR's, columns 3 and 5). As shown the smoothed log R ratio method appear to detect many more cis associations for a given FDR than the CGHseg method.
(XLS)
